# Trypophobia: What Do We Know So Far? A Case Report and Comprehensive Review of the Literature

**DOI:** 10.3389/fpsyt.2018.00015

**Published:** 2018-02-09

**Authors:** Juan Carlos Martínez-Aguayo, Renzo C. Lanfranco, Marcelo Arancibia, Elisa Sepúlveda, Eva Madrid

**Affiliations:** ^1^Department of Paediatrics, Faculty of Medicine, Universidad de Valparaíso, Viña del Mar, Chile; ^2^Interdisciplinary Centre for Health Studies (CIESAL), Universidad de Valparaíso, Viña del Mar, Chile; ^3^Department of Psychiatry and Mental Health, University Psychiatric Clinic, Faculty of Medicine, Universidad de Chile, Santiago, Chile; ^4^Department of Psychology, University of Edinburgh, Edinburgh, United Kingdom; ^5^Biomedical Research Centre (CIB), Faculty of Medicine, Universidad de Valparaíso, Viña del Mar, Chile; ^6^Cochrane Centre, Universidad de Valparaíso, Viña del Mar, Chile; ^7^Department of Paediatrics, Carlos van Buren Hospital, Valparaíso, Chile

**Keywords:** trypophobia, phobic disorders, anxiety disorders, fear, visual perception, biological mimicry, biological evolution

## Abstract

In this article, we describe the case of a girl who suffers from a phobia to repetitive patterns, known as trypophobia. This condition has not yet been recognised by diagnostic taxonomies such as the Diagnostic and Statistical Manual of Mental Disorders. Trypophobia usually involves an intense and disproportionate fear towards holes, repetitive patterns, protrusions, etc., and, in general, images that present high-contrast energy at low and midrange spatial frequencies. It is commonly accompanied by neurovegetative symptoms. In the case we present here, the patient also suffered from generalised anxiety disorder and was treated with sertraline. After she was diagnosed, she showed symptoms of both fear and disgust towards trypophobic images. After some time following treatment, she only showed disgust towards said images. We finish this case report presenting a comprehensive literature review of the peer reviewed articles we retrieved after an exhaustive search about trypophobia, we discuss how this case report contributes to the understanding of this anxiety disorder, and what questions future studies should address in order to achieve a better understanding of trypophobia.

## Introduction

Fear is the normal response to danger, while phobias are characterised by excessive, unconscious, and persistent fear that constantly triggers anxiety. Therefore, in the Diagnostic and Statistical Manual of Mental Disorders (DSM)-5 (1), phobias receive the name of specific phobias and are classified according to their trigger. A particular type of phobia, known as “trypophobia” (2), is described as fear or repulsion to patterns of holes. The word derives from the Greek *trypa* (τρύπα), which means “drilling” or “hole.” Trypophobia is basically referred to by people who suffer from it, through communications such as social networks and personal blogs.

We hereby present a case of an infant who suffers from trypophobia. After obtaining informed consent from her proxy for publishing this report, we hereby report and comment her morbid records, evolution, and response to the treatment. We also present a comprehensive literature review about the condition. Due to the scarce available literature, this case report represents an opportunity to ponder upon the clinical presentation and potential underlying aetiology of this phobia. Finally, we pose some questions about trypophobia for future studies.

## Background

A 12-year-old patient, female and Caucasian, schooled (sixth grade), brought by her mother consulting about her feelings of discomfort when observing or approaching some sorts of surfaces and objects. The patient had a normal pregnancy history and her school performance was outstanding, being above the 90th percentile of her class.

### Family History

The patient currently lives with her mother and a younger sister. Although she has never lived with her father, she visits him twice a month, keeping a close relationship. Her mother suffered from generalised anxiety disorder (GAD) and has additionally developed three depressive episodes successfully treated with sertraline.

### Past Medical History

At the age of 9, the patient consulted a psychiatrist due to physical complaints, e.g., recurrent abdominal pain and nausea. The psychiatrist attributed them to be caused by her parents’ divorce, diagnosing her with separation anxiety disorder. She was treated with 50 mg of daily oral sertraline for 6 months, which yielded a positive response.

### Current Medical History

**Figure 1:**
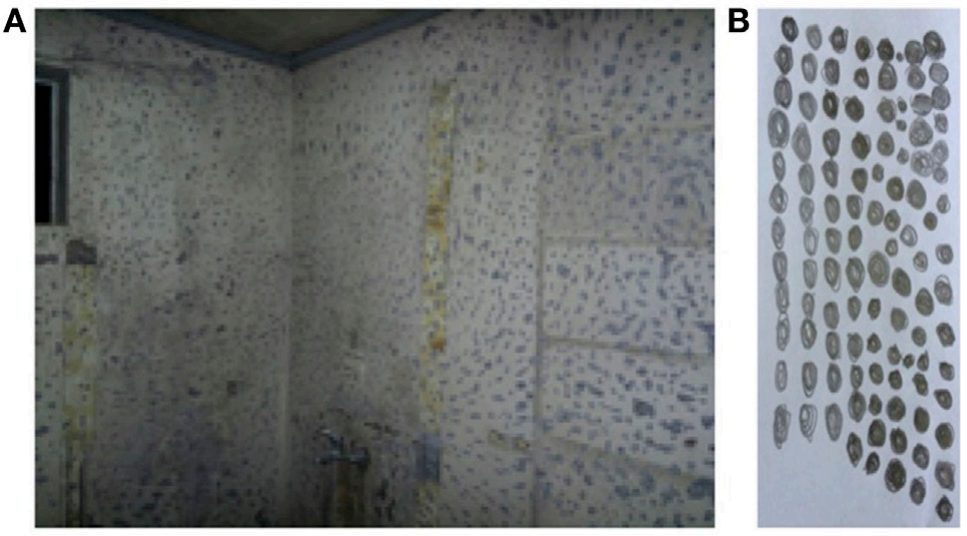
**(A)** Bathroom wall in repair that triggered phobic symptoms according to the patient. **(B)** Drawing made by the patient after being asked: “how would you draw your fear?”

The patient’s symptoms began 3 months before her medical appointment. They included intense, disproportionate, and uncontrollable fears, which were associated with the sight of dotted objects, holes, or particularly bright or conspicuous protruding elements. Such fear was always associated with neurovegetative symptoms, e.g., increased heart and breathing rate, choking, nausea, pallor, dry mouth, sweating, and agitation. She described herself experiencing fear during daily activities, e.g., when looking at sliced or seed-covered bread, *Gruyère* cheese, and clothes with polka dots or animal print. When trying to determine the symptoms’ onset, the mother reported an episode during which her daughter desperately escaped from the bathroom, right after spotting its perforated concrete walls. However, she could not explain neither the situation nor her reaction (Figure 1A). The mother also reported an event when the patient saw a picture of empty honeycomb cells, which yielded in her weeping and anguish, in spite of having never expressed fear to bees before.

The attending psychiatrist kept seeing the patient monthly over the course of a year, where he could determine that her fear was triggered by objects with repetitive clusters. When the patient was asked to draw her fear, she did it as shown in Figure 1B.

While the main complaints revolved around her phobic symptoms, the patient also expressed uncertainty regarding the future, highlighting an exacerbated aversion to change and risk situations, and excessive worries about her academic and social performance, despite the fact that her academic functioning was optimal and her relationship with adults and peers was positive.

Furthermore, she showed a strong pessimistic bias, desire to have everything under control, e.g., closing windows and doors to prevent the entry of thieves, devoting many hours to study, etc. The patient also manifested insomnia and anxious dreams.

Regarding depression and anxiety dimensions, she scored 7 in the Kovacs Questionnaire for Child Depression (3, 4), validated in Chile (a cutpoint of 19 points out relevant depressive symptoms); in the Spence’s Children Anxiety Scale (5, 6), she scored: 55 in panic crisis and agoraphobia, 65 in separation anxiety, 60 in fear of physical harm, 60 in social phobia, 45 in obsessions–compulsions, and 60 in GAD (a cutpoint of 60 is considered “high”). In the Clinical Global Impression Scale of Severity (CGI-S) (7, 8), she obtained a score of 5 (“strongly ill”). Biochemical profile, electroencephalogram, and computed tomography showed no pathological findings.

The psychiatrist diagnosed phobia of repetitive patterns and GAD, discarding any other categorical disorder. After diagnosis, she underwent outpatient therapy with 25 mg of daily oral sertraline in single dose in the morning, increasing to 50 mg after a week, along with cognitive behavioural therapy (CBT).

At the 5-week control, she was asked to assess her phobic symptoms, which she described as a 20% improved when compared to the first consultation. The mother described critical events during that month: first, the girl grasped to her at the supermarket when seeing a set of breads covered with sesame seeds, and second, she reacted similarly when passing by polished stones mounted on a concrete wall. In both events, she showed fear, but without neurovegetative symptoms. Regarding GAD, she showed a substantial improvement on her sleeping disturbances, anxious expectation, and excessive worries; thereby, her behaviour became more suitable to the school obligations she must regularly fulfill. The CGI-S score was 4 (“moderately ill”) and CGI of Clinical Improvement (CGI-I) score was 3 (“slightly better”). Subsequently, sertraline dose was increased to 75 mg.

**Figure 2:**
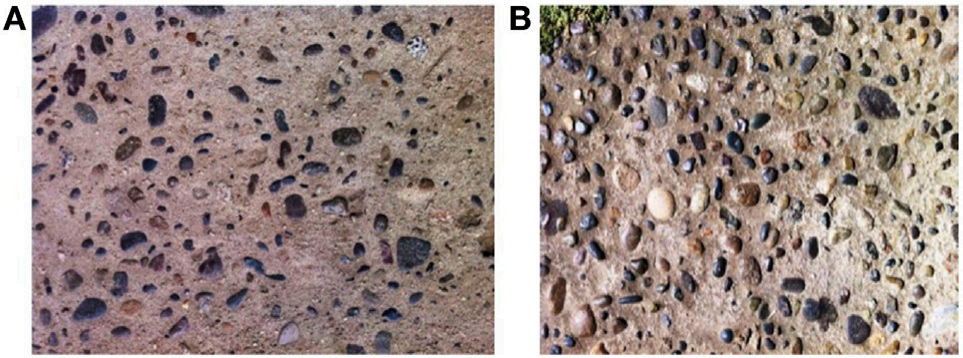
Sidewalks with surface patterns which triggered phobic reactions according to the patient’s mother. **(A)** Sidewalk with stones in the concrete yielding little phobic reaction. **(B)** Sidewalk with stones in the concrete that caused high phobic reaction with neurovegetative symptoms.

**Figure 3:**
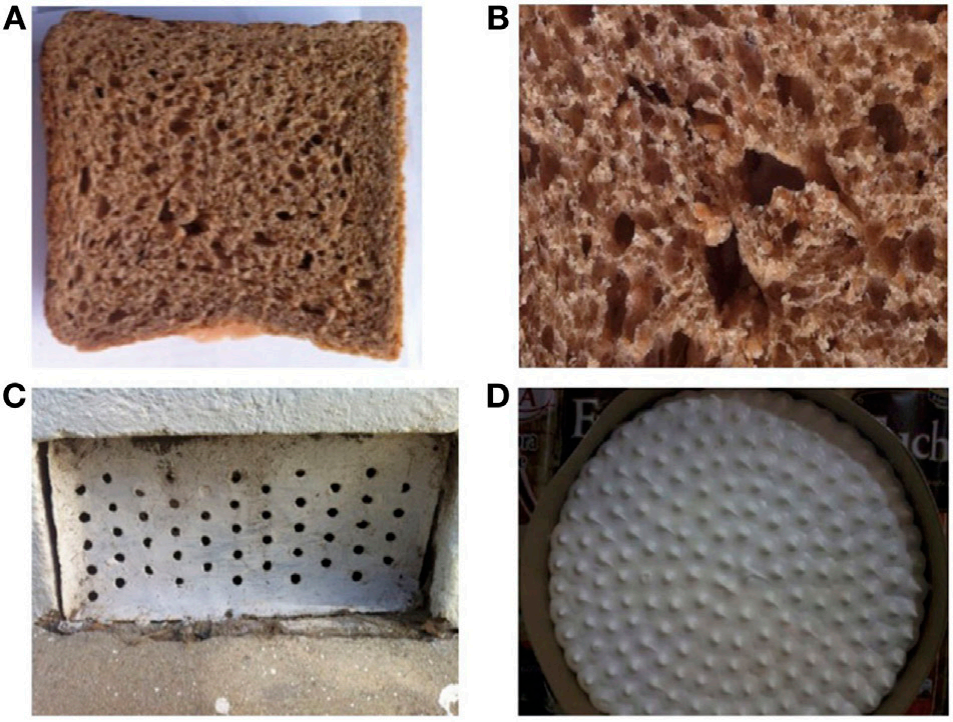
Objects with surface patterns which triggered phobic reactions according to the patient’s mother: **(A,B)** holes in a slice of bread, **(C)** pattern of holes in a piece of metal, **(D)** Sharp spikes on the meringue frosting of a pie.

Nine weeks after starting treatment, the phobia’s intensity had been halved, achieving adequate fear control and facing in a more adequate manner certain situations. The mother provided the health staff with a set of pictures of the objects that had triggered phobic reactions in the patient (see Figures 2 and 3). At that moment, the CGI-S score was 3 (“mildly ill”) and the CGI-I score was 1 (“much better”).

At the last regular medical appointment, the mother referred some situations: since the patient was 4 years old, she has refused certain kinds of foods, throwing tantrums when being forced to eat. The patient referred she felt no fear, but disgust for some kinds of food. In her words: “I could not eat bread with holes, word noodles soup, or drink raspberry juice, because the texture of the seeds generates disgust in me, and I still feel chill when I remember it.” She avoids consuming fruit marmalades due to the feeling of the granules on her tongue, nor has ever eaten strawberries because she perceives them as visually disgusting. During the interview, an image of a strawberry was shown to the patient. She manifested progressive anguish as the image was expanded; the same happened to the image of vents; however, fear was not incipient, although it emerged when the image was amplified.

## Discussion

The understanding of trypophobia is still limited and the number of peer-reviewed articles is low. We searched for publications on PubMed and EMBASE using and combining the free terms: “trypophobia,” “fear*,” “phobi*,” “visual discomfort,” “repetitive patterns,” “holes,” “cluster of holes,” and “stripped pattern.” Only 10 publications met our criteria. They are reviewed below.

### Previous Studies about Trypophobia

“Visual discomfort” was described by Wilkins as an umbrella term describing a spectrum of adverse events triggered by visual stimuli, such as striped pictures, cluster images, repetitive patterns, and even text lines. It is common in individuals suffering from migraine and epilepsy, but it has been mostly studied from the point of view of visual perception rather than the underlying cognitive mechanisms of a phobic phenomenon (9, 10).

Twenty years ago, Rufo (11) described the case of a little girl who was an inpatient in a day-hospital and expressed an extreme terror to holes, describing her paralyzing terror to a repetitive pattern on a surface of a musical instrument. Much later, Cole and Wilkins (2) described trypophobia as a type of visual discomfort provoking an excessive fear when observing patterns of holes. They found that such images possess high-contrast energy at midrange spatial frequencies. It had been reported before that images with such visual properties are often described as uncomfortable by general population (12). In addition, they demonstrated images of highly poisonous animals possess similar spectral features to trypophobic images. Another experiment showed even non-trypophobic individuals are sensitive to this kind of images, some of whom find them aversive. Apparently, there is an innate aversion towards such stimuli.

Le et al. (13) created a 17-item scale to evaluate trypophobic symptoms. This scale, called trypophobia questionnaire (TQ), presents high internal consistency, convergent validity, good test–retest reliability, and very good sensitivity and specificity. They also demonstrated TQ has discriminant validity as its results showed a weak relationship with anxiety scores. They found little evidence to suggest that general anxiety could account for trypophobia. They also ran an experiment, where they showed both clusters of holes and bumps trigger similar levels of discomfort among trypophobic participants. However, the larger the cluster, the higher the discomfort.

A couple of years ago, Chaya et al. (14) further explored the effects of trypophobic aversion. The authors studied whether fear of eye contact or of being gazed at, suffered by people with social anxiety (SA), might be associated with trypophobia (15, 16). SA could have an influence on the degree of discomfort triggered by trypophobic images composed of clusters of human eyes and faces. They found both eyes and faces induce discomfort as the number of images increase. This effect turned out to be strongly related to the SA characteristics, which was also mediated by trypophobia. Notably, the SA feature more strongly predicted discomfort for clusters of eyes on faces compared to isolated eyes.

Imaizumi et al. (17) explored the traits which were predictors of trypophobia proneness, specifically disgust sensitivity, empathic traits, and visual discomfort. They tested whether trypophobia may be an extension of intrinsic disgust for scars, sores, and poisonous animals with scaly or spotty skins (2). They suggest trypophobia proneness may be related to empathic traits, like perspective taking, empathic concern, and personal distress. They found core disgust sensitivity, personal distress, and proneness to visual discomfort may predict TQ scores as dependent variable. However, the determination coefficients in their regression models were relatively low, suggesting other psychological factors may contribute to trypophobic scores as well (17).

Vlok-Barnard and Stein (18) studied whether trypophobia resembles more specific phobias or obsessive-compulsive disorder. They studied socio-demographic factors, severity of psychological distress, etc., and whether trypophobic stimuli trigger either fear or disgust more often. They found most of the participants who suffered from trypophobia fulfilled the DSM-5 criteria for specific phobia, experiencing disgust rather than fear when confronted with clusters of holes, but not meeting the distress or impairment clinical criterion. Only a small percentage fulfilled the DSM-5 criteria for obsessive–compulsive disorder. Notably, 60.5% reported mostly disgust when confronted with clusters of holes, while 11.8% reported only disgust, 5.1% reported mostly fear, while 1% experienced only fear, and 21% experienced the same amount of fear and disgust. Other findings refer to trypophobia being more prevalent in women, being chronic and persistent, and having common co-morbid psychiatric diagnosis, such as major depressive disorder (MDD) and GAD.

Recently, Can et al. (19) questioned whether trypophobia is really a phobia, given that previous reports showed people commonly feel disgust rather than fear when confronted to trypophobic images. Hence, if trypophobia does not involve fear in most cases, it might not be a specific phobia. They studied the discomfort related to trypophobic features and whether it is grounded in their visual features or on associated threat of venomous animals in young children, as their experience perceiving non-visual properties of venomous animals is very limited. Pre-schoolers do not show fear of snakes or spiders, for instance. To test this, they showed them both trypophobic photos of venomous (snakes) and of non-venomous animals (sea stars). They asked the pre-schoolers to rank the images according to how much they liked them. Next, they performed an implicit association test to measure the strength of the associations between mental representations of concepts at a non-conscious level. The children were asked to categorize images of snakes, sea stars, and holes, reporting a negative valence towards trypophobic images and venomous animal photos, and a neutral attitude towards venomous animals without trypophobic patterns. However, the researchers did not find a significant association between trypophobic features and threat of venomous animals at a non-conscious level. In summary, the unconscious association between clusters of holes and threat of venomous animals was not present. If there is an association between trypophobic images and threat of venomous animals due to evolution, it would appear later in life.

Recently, Kupfer and Le (20) posed trypophobia could be due to aversion to ectoparasites and skin-transmitted pathogens in humans. There is no evidence that poisonous animals posed a significant threat in ancestral times, while parasitism and infectious disease certainly did. Most virulent and deadly diseases from the past involved irregular clusters of pustules or roughly circular shapes on human skin. To test this, they measured disgust and fear, including emotion responses and open-ended descriptions of what participants experienced after looking at trypophobic images of body parts or ectoparasites and other small animals, and at cluster images with disease-irrelevant properties such as drilled holes. They also included non-trypophobic images as controls. They found that both trypophobic and control participants exhibited high levels of aversion towards disease-relevant cluster images. However, only trypophobic participants exhibited high levels of aversion towards disease-irrelevant cluster images. Since these results could neither be explained by differences in sensitivity nor by differences in neuroticism, they interpreted this as consistent with their hypothesis that trypophobia is an over generalised aversion towards cluster stimuli that indicates a parasitic and infectious disease threat. Additionally, they found disgust elicited by both disease-irrelevant and disease-relevant stimuli was significantly higher than fear in trypophobic participants.

Sasaki et al. (21), further explored how the spatial frequency of a stimulus can be related to trypophobic discomfort. It was previously reported by Cole and Wilkins (2) that images that contain high-contrast energy at midrange spatial frequencies caused discomfort. However, the role of spatial frequency was not tested in detail. For this reason, Sasaki et al. (21) ran a set of three experiments to test what specific spatial frequencies are most important when causing visual discomfort in trypophobic people. They employed the same stimuli previously used by Le et al. (13) and found that both low and midrange spatial frequencies, together or separately, are essential for causing trypophobic discomfort with images of holes. This would be especially remarkable when comparing the discomfort of people with high trypophobic scores (TQ score) against people with low scores. In other words, trypophobic discomfort was able to predict visual discomfort only for trypophobic images showing low or midrange spatial frequencies.

More recently, Yamada and Sasaki (22) proposed a hypothesis based on the results obtained by previous studies. They argued that people experience negative emotions when looking at trypophobic objects because they probably associate their surfaces with skin diseases. Thus, trypophobic stimuli would trigger an avoidance reaction towards pathogens. This hypothesis, called involuntary protection against dermatosis (IPAD) hypothesis was tested through one study, an online survey with 856 participants. They were asked to assess the discomfort caused by the images on a 11-point scale. Crucially, the researchers found that participants with a history of skin problems reported experiencing significantly higher discomfort than those without skin problems history. These findings were confirmed in a subsequent replication with 690 new participants who completed the survey. The authors argue that these results support the IPAD hypothesis while also suggesting the presence of a cognitive mechanism of dermatosis-avoidance. Therefore, a causal relationship between trypophobia and skin diseases remains unclear, but it is suggested.

In summary, the evidence so far suggests people show aversion towards images with high-contrast energy both at low (21) and at midrange spatial frequencies (2). All trypophobic images share such features. Crucially, trypophobic people would be especially sensitive to such features, exhibiting high levels of fear or disgust when presented with them (2). A questionnaire was developed to measure trypophobic symptoms, and it was shown that these are no explained by GAD symptoms (13). Additionally, both trypophobia and SA have demonstrated to contribute to the discomfort that some people experience when gazed at by many people (14). Furthermore, several psychological traits such as visual discomfort, disgust sensitivity, and empathic traits predict the discomfort caused by trypophobia (17). Likewise, it has been determined that trypophobia is more prevalent in women, being chronic and persistent, and its most common co-morbid diagnoses are MDD and GAD. Furthermore, trypophobia would commonly involve a family history of the disorder as well (18). Moreover, the hypothesised association between poisonous animals and trypophobia has not been established (19). There might be an association between aversion to irregular clusters of pustules or roughly circular shapes on human skin and trypophobia (20), but such an association is still unclear. A very recent theory proposed by Yamada and Sasaki (22) points in this direction. Originally, Cole and Wilkins (2) speculated that trypophobic stimuli would yield discomfort because they resemble poisonous animals, while Kupfer and Le (20) proposed that the reason behind this phenomenon would be that such images are similar to scars and sores thus explaining the disgust experienced by trypophobic people when looking at them. Yamada and Sasaki (22) proposed that trypophobia would entail an involuntary reaction based on an avoidance response towards dermatitis-related stimuli. The main articles are summarised in Table 1.

**Table 1:** Summary of studies, instruments, and contributions about trypophobia.

Reference	Type of sample	Instruments employed	Main findings and contributions
Rufo (11)	Case report	Psychiatrist’s report	First available description about a girl who expressed panic when facing any image of repetitive patterns, specially holes

Cole and Wilkins (2)	General population of adults and adults who claim to suffer from trypophobia	Rating scales, spectral analysis	All trypophobic images possess high-contrast energy in midrange spatial frequencies, a feature also shared by images of poisonous animals. People in general experience discomfort when looking at trypophobic images

Le et al. (13)	General population of adults and adults who claim to suffer from trypophobia (Facebook group)	Rating scales, questionnaire	The construction of the TQ and the presentation of its psychometric properties. They confirm that people who suffer from high levels of trypophobia are more sensitive to images with high-contrast energy in midrange spatial frequencies. Both images of holes and bumps can trigger trypophobic symptoms. Also, the bigger the cluster, the higher the trypophobic response

Chaya et al. (14)	General population of adults (recruited online)	TQ, Liebowitz Social Anxiety (SA) Scale, Discomfort Rating Score	SA has a significant indirect effect on the discomfort associated with eye clusters, which was mediated by trypophobia. The same happens with clusters of faces. The results suggest both SA and trypophobia contribute to the discomfort some people experience when gazed by many people

Imaizumi et al. (17)	General population of adults (recruited online)	TQ, Disgust Scale-Revised, Interpersonal Reactivity Index	Trypophobia proneness is predicted by core disgust sensitivity, personal distress, and proneness to visual discomfort

Vlok-Barnard and Stein (18)	Adults who claim to suffer from trypophobia (Facebook group)	Self-report questionnaire, Kessler Psychological Distress Scale, Sheehan Disability Scale, and items from Zohar–Fineberg Obsessive–Compulsive Screen and Diagnostic and Statistical Manual of Mental Disorders -5 criteria for Specific phobias	Trypophobic symptoms are chronic and persistent and cause significant distress. The most common co-morbidity diagnoses are major depressive disorder and generalized anxiety disorder. The most common trypophobic symptom is disgust rather than fear when confronted with trypophobic images

Can et al. (19)	4-year-old children randomly recruited	Self-report, categorization task	Trypophobic stimuli are associated with discomfort in children due the visual features of said stimuli. The results suggest that such discomfort is due to an instinctive response to the stimuli visual features rather than the result of a learned but non-conscious association with venomous animals

Kupfer and Le (20)	General population of adults and adults who claim to suffer from trypophobia (Facebook group)	Fear and disgust self-report scales, Three Domain Disgust Scale, Neuroticism subscale (from Big Five Inventory)	Both people who suffer from trypophobia and who did not report aversion towards disease-relevant cluster stimuli, but only the trypophobic group reported aversion towards objectively harmless cluster stimuli that had no relevance to disease. Aversive responses were predominantly based on disgust

Sasaki et al. (21)	General population of adults	TQ, Discomfort scale	Trypophobic discomfort can be caused both by mid- and low-frequency visual components

Yamada and Sasaki (22)	General population of adults (recruited online)	Discomfort surveys	Discomfort evoked by trypophobic images is higher amongst participants with history of skin problems

### What Does This Case Report Contribute for Future Research?

First, most trypophobic people show disgust instead of fear as main symptom. The most common response in phobias is disproportionate fear, which this patient suffered along with disgust before treatment. After treatment, she communicated experiencing mostly significant disgust. This could mean trypophobic symptoms make the individual vulnerable to suffer from anxiety due to negative reinforcements in operant conditioning, e.g., individuals more sensitive to trypophobic images may try to avoid them and, thus, avoid the concomitant discomfort. This could increase anxious expectation with time, as it appears to happen with specific phobias (23). Future studies should determine what reaction (disgust or fear) is related to more severe symptoms and whether this is relevant for treatment. Second, future studies should explore differences in treatment response among trypophobic people who exhibit mainly fear, and people who exhibit mainly disgust. Third, future studies should determine whether trypophobia may cause significant distress when presented without other diagnoses. Finally, future studies should explore the nature of other sensory perceptions of repetitive patterns, considering that our patient also expressed aversion towards tasting foods and liquids with trypophobic-like surfaces, which suggests that trypophobic symptoms might not be exclusive for visual stimuli. If this is a fact that encompasses a significant amount of people with high trypophobic scores (TQ scores), then the nature of trypophobia would go beyond the scopes of works reviewed here.

It has been reported sertraline plus CBT would achieve greater effects in children anxiety (24). A long-term treatment was suggested since about half of the children who suffer from anxiety disorders relapse during the acute stage (25). Treatment with sertraline was effective both for phobic and anxiety symptoms in this case, corroborating its efficacy. However, proper clinical trials are of course desirable.

The origin of phobias has been attributed to evolutionary principles (26–29), to classical conditioning (30–32), and to beliefs and cognitive biases related to objects, situations, and attentional focus (33–36). We propose that trypophobia may be caused by both evolutionary factors and operant conditioning, where the natural reaction acquired through evolution is disgust towards trypophobic images. This disgust response may thus develop fear over time and then turn into a specific phobia due to negative reinforcement by avoidance as avoidance behaviour has shown to contribute to the persistence of fear and the amplification of anxiety over time (37). This way, anxiety, and anxious expectation would grow with time, adding symptoms of fear on top of the innate symptoms of disgust. Our theory does not deal with the origin of trypophobic disgust. However, it provides a framework to understand how a natural response of discomfort found in both clinical and healthy populations can become a specific phobia with all what a specific phobia usually entails. Nevertheless, a few sensible questions arise. What is the role of disgust in the development of a specific phobia? Can disgust help developing fear to trypophobic stimuli over time? Even though several specific phobias present both disgust and fear as key symptoms (38, 39), the role of disgust in trypophobia symptoms needs to be explored further. Finally, whether visual discomfort towards trypophobic images comes from an innate aversion towards poisonous animals (2, 19), scars, sores and illnesses (20), or dermatosis signs (22) does not explain how an innate response of disgust can turn into a specific phobia. This is an especially crucial matter for clinical research.

### Limitations and Considerations

The approach taken in this case report presents some limitations that need to be explained and some considerations that should be taken. First, as described before, Le et al. (13) developed TQ, a questionnaire to evaluate trypophobic symptoms. Unfortunately, we could not use this instrument with the patient of this report because at the time of her evaluation such instrument was not available yet. Nonetheless, we stress the importance of TQ to measure trypophobic symptoms in a more objective way as much as its usefulness as an instrument to measure response to treatment and therapeutic progress.

Second, the fact that the patient responded positively to sertraline and CBT as treatments does not mean that sertraline should be necessarily regarded as a standard treatment method for trypophobic symptoms. The rationale for choosing sertraline here lies on the fact that the patient’s mother suffered from GAD and depressive episodes in the past, having yielded a very positive response for both ailments when treated with sertraline. Taking into consideration that, of course, both mother and daughter share a strong genetic background, and also the fact that GAD and trypophobia are both anxiety disorders with allegedly common underlying neurobiological mechanisms, it seemed justified to prescribe sertraline and CBT to the patient.

Finally, it is necessary to state to what extent we can take this case report as a representative case of trypophobia. To answer this question, we first need to answer what is the relation between the trypophobic symptoms presented by the patient and trypophobic disgust and fear described in previous articles. As described before, the patient has exhibited aversive discomfort towards trypophobic stimuli from very early age. This discomfort became worse with time until the phobic symptoms triggered by trypophobic images began to significantly affect her daily life. The similarities between this case report and previous descriptions in the literature lie upon the discomfort presented towards trypophobic stimuli, as defined by Cole and Wilkins (2) and Le et al. (13). The phobic stimuli described by the patient and her mother match the typical trypophobic stimuli usually described as discomforting by trypophobic population. However, there are a few differences that need to be stated here. For instance, the patient was diagnosed with phobia of repetitive patterns (i.e., trypophobia) and GAD, which means that several of her symptoms did not correspond exclusively to trypophobic symptoms but to a higher general level of anxiety. As a co-morbid diagnosis, it could have affected the presentation of trypophobia, but GAD itself could not explain the nature and presentation of trypophobic symptoms because of how specific the latter were. Even so, this case report describes a patient who suffers from trypophobic symptoms that effectively caused both persistent, excessive, and unreasonable disgust and fear. Future studies and case reports are needed in order to determine the limit between trypophobic discomfort as an aversive response and trypophobia as a clinically valid anxiety disorder.

## Concluding Remarks

Trypophobia has been described as a phobia to images with high-contrast energy at low and midrange spatial frequencies, such as holes and repetitive patterns, and two theories, both evolutionary, have been posed: trypophobia could be a product of evolution, an aversion to poisonous animals that possess high-contrast energy at midrange spatial frequencies in their skin, or due to an aversion to clusters of pustules or roughly circular shapes on human skin, thus helping humans to avoid ectoparasites and infectious diseases. A third and more recent theory about trypophobia as an involuntary reaction towards dermatosis has also been posed. Neither of these theories has been supported by substantial evidence yet. However, multiple aspects of trypophobia have been partially determined, such as socio-demographic variables, clinical features, co-morbidities, levels of distress, associated psychological traits, and visual features of the stimuli. Moreover, a symptom scale has been developed and validated for the study of trypophobia. One of the mysteries that have not been solved yet is that there are people who express disgust, while others express fear or both to trypophobic images. Assuming trypophobia is due to evolution, then it makes sense to ask why some people react with disgust while others react with fear? This problem is key for the clinical understanding of trypophobia as a specific phobia and it has not been answered yet.

## Ethics Statement

This study involves a case report, for which a written informed consent was obtained from the parents of our patient in order to publicly describe her case, in accordance with CIOMS guidelines and the Declarationof Helsinki.

## Author Contributions

JM-A: conception of the work, acquisition and interpretation of the data, critical revision of the work, and final approval of the version to be published. RL, MA, ES, and EM: conception of the work, interpretation of the data, critical revision of the work, and final approval of the version to be published.

## Conflict of Interest Statement

The authors declare that the research was conducted in the absence of any commercial or financial relationships that could be construed as a potential conflict of interest.
